# Smokers have increased risk of soft-tissue complications following primary elective TKA

**DOI:** 10.1007/s00402-023-04771-8

**Published:** 2023-01-13

**Authors:** Moritz Starzer, Maria Anna Smolle, Ines Vielgut, Georg Hauer, Lukas Leitner, Roman Radl, Reinhard Ehall, Andreas Leithner, Patrick Sadoghi

**Affiliations:** 1 Department of Orthopedics and Trauma, Medical University of Graz, Graz, Austria; 2 Department of Orthopedics and Trauma, LKH Hochsteiermark, Tragösserstraße 1, 8600 Bruck an der Mur, Austria; 3 Department of Orthopedics and Orthopedic Surgery at the LKH Südsteiermark, Radkersburg, Austria

**Keywords:** Smoking, Smokers, Former smokers, Never smokers, Total knee arthroplasty, Revision, Complication, Outcome, TKA

## Abstract

**Introduction:**

Smoking has been associated with numerous adverse outcomes following surgical procedures. The purpose of this study was to investigate, whether smoking status at time of surgery influences the outcome of primary TKA.

**Materials and methods:**

Six hundred and eighty-one patients who underwent primary TKA between 2003 and 2006 were included in the study. Smoking status was defined as current, former, and never smoker. Complications leading to revisions were assessed until 17 years of follow-up. Functional outcome was evaluated using clinical scores: Western Ontario and McMaster Universities Osteoarthritis Index (WOMAC), Visual Analogue Scale (VAS) for pain, Short Form-12 Physical and Mental Component Summaries (SF-12PCS/MCS), and Knee Society Function and Knee Score (KSFS and KSKS).

**Results:**

At a mean follow-up of 95 months (± 47 months), 124 complications led to revision surgery. Soft-tissue complications (OR, 2.35 [95% CI 1.08–5.11]; *p = *0.032), hematoma formation (OR, 5.37 [95% CI 1.01–28.49]; *p = *0.048), and restricted movement (OR, 3.51 [95% CI 1.25–9.84]; *p = *0.017) were more likely to occur in current smokers than never smokers. Current smokers were more likely to score higher at KSFS (*p < *0.001) and SF-12PCS (*p = *0.0197) compared to never smokers. For overall revision, differences were noted.

**Conclusion:**

Current smoking increases risk of soft-tissue complications and revision after primary TKA, especially due to hematoma and restricted movement. Smoking cessation programs could reduce the risk of revision surgery.

## Introduction

Tobacco smoking has been identified as a risk factor for adverse postoperative outcomes, including wound-related complications, surgical-site infection, and cardiopulmonary complications [1, 2]. Hawn et al. revealed an increased risk of postoperative complications for smokers regardless of surgical specialty and case complexity [1]. Total knee arthroplasty (TKA) is a frequently performed procedure with approximately 700.000 implanted in the USA yearly, with estimations suggesting a continuing increase [3–5]. Previous studies have confirmed the hypothesis that tobacco smoking increases the risk of overall postoperative complications after elective orthopedic surgery and TKA [6–8]. Regarding prosthesis-related complications, Singh et al. discovered smoking as a risk factor for deep infection and implant revision after primary TKA and THA [9]. Lim et al. have concluded a higher risk of earlier revision in smokers [10]. However, Matharu et al. did not find an increase in long-term revision rates [11]. Despite Matharu et al. revealing no clinically significant differences in postoperative patient-reported outcome measures (PROMs) between smokers, former smokers, and never smokers, literature on PROMs after TKA is scarce [11].

The literature describes a decrease in smoking and alcohol consumption in the initial 12-month post-THA and TKA [12]. The cited literature has a follow-up of 30 days up to 10 years, whereas long-term follow-up over 15 years is missing [6–11].

The aim of this study was to identify whether smoking status (active, former, never smoker) at time of surgery influences the outcome of TKA in terms of prosthesis-related complications and postoperative PROMs. The hypothesis was that current smokers have a higher risk of complications, revision surgery, and decreased outcome.

## Materials and methods

### Patient population

**Fig. 1:**
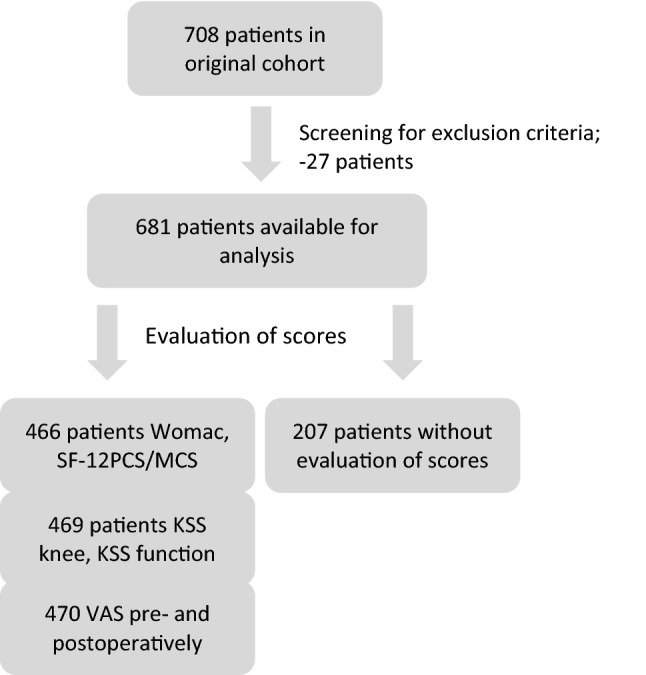
Flowchart illustrating the patient selection process

For this retrospective analysis, an adjusted preexisting study cohort was evaluated, consisting of patients having received primary TKA [13]. Regular follow-ups were performed by clinical examination at two supra-regional departments. Primary surgery had been performed between 2003 and 2006 by experienced orthopedic surgeons, resulting in an observation period of up to 17 years. Seven hundred and eight (708) patients were initially included. Exclusion criteria were primary TKA before 2003, patients having received revision TKA during the study period, no smoking status at primary surgery available, and death. After checking for exclusion criteria, data of 681 patients were attained for statistical analysis regarding postoperative complication risk (illustrated in Fig. 1). Due to the retrospective study design and pre-selected study cohort, not all patients have been evaluated regarding PROMs before gathering the data, resulting in 466 cases with WOMAC, SF-12PCS, SF-12MCS, 467 with KSFS, 469 with KSKS, and 470 with VAS.

The smoking status is based on the smoking behavior within the year prior to assessment. Having smoked regularly counted as current smoker, having stopped smoking within the year prior to assessment or before was defined as former smoker, and never having smoked regularly counted as never smoker. Pack history, i.e., cigarette smoking exposure rate was not available.

Primary outcome was any implant-specific complication for which revision surgery became necessary. Complications were divided into soft-tissue complications (wound dehiscence, restricted movement, defined as full extension or flexion of at least 90 degrees 6 weeks after implantation, hematoma, and infection) and mechanical complications (aseptic loosening, periprosthetic fracture, wear, and dislocation) [14]. The secondary outcome was assessment of the Knee Society Function Score (KSFS) and Knee (KSKS) Score, Western Ontario and McMaster Universities Osteoarthritis Index (WOMAC), and Short Form 12 Physical and Mental Health Composite Scores (SF-12PCS and SF-12MCS) at last follow-up. Pain Visual Analogue Scale (VAS) pre- and postoperatively facilitated pain evaluation and improvement [15–19]. Additionally, the mean reduction in VAS pre- to postoperatively was calculated.

Further variables were age at time of surgery, time from primary TKA to revision for implant-related causes, and gender differences regarding revision risk.

This study was conducted in compliance with recognized international and accepted ethical, scientific and medical standards and approved by the local ethics committee (26-527 ex 13/14).

### Statistical analysis

Clinical and demographic characteristics are described by summary statistics. Discrete variables are presented as proportions and percentages. Means and medians with corresponding standard deviations and interquartile ranges (IQRs) are presented. For the comparison of discrete variables, the Chi-squared test for proportions was used. For analysis and comparison of mean values of binominal data and continuous data, a two-sample, unpaired *t* test was used. Logistic regression was performed to assess the odds ratios (ORs) of risk factors for complications. A *p* value of less than 0.05 was considered statistically significant.

## Results

### Patient characteristics

For the 681 patients, mean follow-up was 95 months (± 47 months). Table 1 gives an overview of the demographic characteristics. Of 681 patients, 478 (70.2%) were female and 203 (29.8%) were male. Current smokers, former smokers, and never smokers had shares of 46 (6.8%), 39 (5.7%), and 596 (87.5%), respectively. In relation to gender, 56.5% (26) of current smokers were female and 43.5% (20) were male. Analysis showed a higher likelihood of current smokers to be male than female (*p = *0.010).

**Table 1:** Average age at time of surgery in relation to smoking status

	Active smoker (*n = *46) SD ( ±)	Never smoker (*n = *596) SD ( ±)	*p*	Former smoker (*n = *39) SD ( ±)	Never smoker (*n = *596) SD ( ±)	*p*	Active smoker (*n = *46) SD ( ±)	Former smoker (*n = *39) SD ( ±)	*p*
Age surgery	57.0 (± 9.7)	69.6 (± 8.2)	** < 0.001**^**b**^	63.3 (± 7.7)	69.6 (± 8.2)	** < 0.001**^**b**^	57.0 (± 9.7)	63.3 (± 7.7)	**0.015**^**b**^

^a^Chi-squared test

^b^Two-sample *t* test with equal variances, age surgery mean age at time of surgery, bold statistically significant *p* value (< 0.05)

Mean age at time of surgery was 68.4 (± 8.9) years. In active smokers and former smokers, the mean age at time of surgery with 57.0 years (*p < *0.001) and 63.3 years (*p < *0.001) was significantly lower compared to never smokers with 69.6 years.

In total, 126 (18.5%) complications were observed, of which 57 (45.2%) were mechanical and 69 (54.8%) were soft-tissue complications, leading to 124 (18.2%) revisions. Median time from primary TKA to revision was 25 months (IQR: 12–51 months). A revision was necessary less than 12 months after surgery for 32 (25.8%) patients, and after greater or equal than 12 months for 92 (74.2%) patients. For overall revision likelihood (i.e., mechanical and soft-tissue complications combined), no statistically significant difference between the different smoking status groups was found. However, current smokers (13/46, 28.3%) were per tendency at higher risk for revision surgery than never smokers (103/596, 17.3%; *p = *NS). For former smokers compared to never smokers, no significant difference could be shown (8/31, 20.5% vs. 103/596, 17.3%; *p = *NS).

Soft-tissue complications were significantly more common in current smokers (19.6% vs. 9.4%; *p = *0.028) than never smokers (OR 2.35 [95% CI 1.08–5.11]; *p = *0.032; Table 2). In terms of individual soft-tissue complication risk, active smokers had a higher likelihood of developing hematoma (4.4% vs. 0.8%; *p = *0.027; OR, 5.37 [95% CI 1.01–28.49]; *p = *0.048) and restricted movement (10.9% vs. 3.4%; *p = *0.011; OR 3.51 [95% CI 1.25–9.84]; *p = *0.017) than never smokers. The smoking status groups shared a similar revision risk regarding mechanical complications. Former smokers neither had an increased nor decreased revision risk compared to never and current smokers.

**Table 2:** Odds ratios of smoking status regarding complications at a mean follow-up of 95 months (± 47 months) after implantation of primary total knee arthroplasty (TKA)

	Never smokers (*n = *596) vs. active smokers (*n = *46)		Never smokers (*n = *596) vs. former smokers (*n = *39)		Active smokers (*n = *46) vs. former smokers (*n = *39)	
	OR (95% CI)	*p*^a^	OR (95% CI)	*p*^a^	OR (95% CI)	*p*^a^
ST comp	2.35 (1.08–5.11)	**0.032**	1.10 (0.38–3.21)	ns	0.47 (0.13–1.67)	ns
Mech. comp	1.06 (0.37–3.10)	ns	1.28 (0.44–3.84)	ns	1.2 (0.28–5.15)	ns
AL	0.96 (0.22–4.2)	ns	2.41 (0.8–7.2)	ns	2.51 (0.43–14.5)	ns
Infection	1.19 (0.27–5.21)	ns	2.17 (0.62–7.61)	ns	1.83 (0.29–11.6)	ns
RM	3.51 (1.25–9.84)	**0.017**	0.76 (0.1–5.8)	ns	0.22 (0.02–1.93)	ns
PF	1.01 (0.13–7.8)	ns	–^b^	–^b^	–^b^	–^b^
Wear	1.36 (0.2–13.35)	ns	–^b^	–^b^	–^b^	–^b^
WD	–^b^	–^b^	–^b^	–^b^	–^b^	–^b^
Hematoma	5.37 (1.01–28.49)	**0.048**	–^b^	–^b^	–^b^	–^b^
Luxation	–^b^	–^b^	–^b^	–^b^	–^b^	–^b^

*OR* odds ratio, *CI* confidence Interval, *ST compl.* soft-tissue complication, *mech. compl.* mechanical complication, *AL* aseptic loosening, *RM* restricted movement, *PF* periprosthetic fracture, *WD* wound dehiscence; *ns* not significant *p* value

^a^Logistic regression, bold statistically significant *p* value (< 0.05)

^b^Odds ratios not calculable due to too less category samples

PROMs postoperatively and VAS pre- and postoperatively were gathered for 466 (WOMAC, SF-12PCS, SF-12MCS), 467 (KSFS), 469 (KSKS), and 470 (VAS) patients. Statistical analyses and different absolutes among the scores are shown in Table 3. Current smokers were more likely to score higher at KSFS (*p < *0.001) and SF-12PCS (*p = *0.0197) compared to never smokers. Additionally, they reported higher pain ratings preoperatively (*p = *0.0031) than never smokers. The remaining scores were similar irrespective of smoking status.

**Table 3:** Mean clinical scores in relation to smoking status at a mean follow-up of 95 months {± 47 months) after implantation of primary total knee arthroplasty {TKA)

	Active smoker *n*^b^ SD ( ±)	Never smoker *n*^a^ SD ( ±)	*p*^c^	Former smoker (*n = *30) SD ( ±)	Never smoker *n*^a^ SD ( ±)	*p*^c^	Active smoker *n*^b^ SD ( ±)	Former smoker (*n = *30) SD ( ±)	*p*^c^
WOMAC	84.0 (± 16.5)	80.1 (± 15.5)	ns	86.2 (± 13.4)	80.1 (± 15.5)	ns	84.0 (± 16.5)	86.2 (± 13.4)	ns
KSKS	84.8 (± 14.2)	82.8 (± 15.8)	ns	87.4 (± 15.7)	82.8 (± 15.8)	ns	84.8 (± 14.2)	87.4 (± 15.7)	ns
KSFS	79.4 (± 24.2)	64.1 (± 26.0)	** < 0.001**	72.1 (± 22.2)	64.1 (± 26.0)	ns	79.4 (± 24.2)	72.1 (± 22.2)	ns
VAS pre	8.3 (± 1.2)	7.6 (± 1.4)	**0.0031**	7.6 (± 1.1)	7.6 (± 1.4)	ns	8.3 (± 1.2)	7.6 (± 1.1)	ns
VAS post	2.2 (± 2.0)	1.9 (± 2.0)	ns	1.3 (± 1.7)	1.9 (± 2.0)	ns	2.2 (± 2.0)	1.3 (± 1.7)	ns
VAS diff	6.0 (± 1.8)	5.6 (± 2.3)	ns	6.3 (± 2.1)	5.6 (± 2.3)	ns	6.0 (± 1.8)	5.6 (± 2.3)	ns
SF-12PCS	41.2 (± 10.9)	36.8 (± 10.4)	**0.0197**	40.4 (± 10.5)	36.8 (± 10.4)	ns	41.2 (± 10.9)	40.4 (± 10.5)	ns
SF-12MCS	53.2 (± 10.5)	52.8 (± 10.9)	ns	54.8 (± 10.3)	52.8 (± 10.9)	ns	53.2 (± 10.5)	54.8 (± 10.3)	ns

^a^*n = 400* WOMAC; *n = 401* KSS function; *n = 402* SF-12PCS, SF-12MCS; *n = 403* KSS knee; *n = 404* VAS pre., VAS post., VAS diff

^b^*n = 34* SF12PCS, SF12MCS; *n = 36* WOMAC, KSS knee, KSS function, VAS pre. VAS post., VAS diff.; *ns* not significant *p* value

^c^Two-sample *t* test with equal variances, bold statistically significant *p* value (< 0.05)

## Discussion

The most important findings of this study were a significantly higher soft-tissue complication rate for current smokers in comparison with never smokers. Current smokers were more likely to undergo revision surgery due to restricted movement and hematoma as compared with never smokers.

Earlier findings were an increased overall revision risk for active smokers against never smokers after primary elective TKA [1, 7–10]. In contrast to the existing evidence [7, 8, 20], we observed the risk of infection not significantly increased in active smokers compared to never smokers. Furthermore, former smokers did not have an overall increased revision likelihood compared to never smokers, as supported by other investigations [10]. We found current smokers (57 ± 9.7 years) and former smokers (63.3 ± 7.7 years) to undergo primary TKA surgery 12.6 years (± 8.2; *p < *0.001) and 6.3 years (± 7.7; *p < *0.001) earlier than never smokers (69.6 ± 8.2 years), which might be due to faster biological aging of smokers in contrast to non-smokers [21] or due to smokers reporting higher musculoskeletal pain ratings, therefore needing an earlier definitive surgical treatment [22].

Some studies have demonstrated a beneficial effect of smoking cessation prior to surgical interventions in general [23, 24]. The results of this study are supported by earlier findings, that smoking is major contributor to delayed wound healing and consecutively also infection [25, 26]. Smoking seems to have an all-or-nothing effect, with several studies suggesting to quit smoking rather than reduce the number of cigarettes per day [27, 28]. Studies have found cessation to be tremendously beneficial before the age of 40, almost eliminating the risk of losing a life-decade, and to be beneficial after the age of 70 [29, 30]. This evidence supports our finding that former smokers have similar complication risks than never smokers and emphasizes the beneficial effects of smoking cessation.

Apart from smoking status, the ASA status, the surgeon’s expertise, case complexity, the hospital environment, and other patient factors (comorbidities) may be important confounding variables, for which we were not able to do adjusted analyses due to a small sample size. Regarding comorbidities, the authors were unable to gather enough precise information to adequately include an analysis. A differentiation in preexisting disease and disease having developed postoperatively was not possible, wherefore proper interpretation would not have been meaningful. The importance of ruling out confounding bias should be emphasized, but the similar results of previous papers support our findings although not having been able to adequately rule out confounding bias.

This study has revealed higher revision rates (18.2%) than mentioned in the previous literature with 6.45% at 5 years and 3–12.9% at 10 years [31, 32]. This could be attributed to a longer follow-up of up to 17 years, a combination of short- and long-term complication-based revisions or an inclusion of complications as defined by Goslings and Gouma and Sokol and Wilson [14, 33]. The higher rate of soft-tissue complications than reported in previous investigations [31, 32] could be explained by smoking being a major cause of soft-tissue complications and a smoking prevalence of 25% in Austria [25, 26].

Previous investigations have reported an age difference between smokers and never smokers regarding primary TKA [7, 9, 20, 34], indicating a particular need to investigate whether smoking should be defined as an independent risk factor of knee osteoarthritis. We did not collect information about the Kellgren-Lawrence classification of individual patients. Therefore, we cannot say whether active smokers had minor or more severe grades of osteoarthritis possibly leading to TKA.

Interestingly, our secondary outcomes resulted in current smokers scoring significantly better at KSFS and SF-12PCS and reporting higher pain ratings prior to surgery. Active smokers score 15.3 points higher than never smokers for KSFS and 4.4 points higher for SF-12PCS. The minimally clinically important difference (MCID) for primary TKA was found to be 9 points for KSKS and 10 points for KSFS [35]. For the SF-12PCS, it was 4.5 and 4.8 points for the pain relief and function sections, respectively [36], rendering these findings as not clinically important. However, active smokers seem to have a clinically better functional outcome than never smokers. Previous research by Matharu et al. found no clinically important differences in patient-reported outcome measures between active smokers, former smokers, and never smokers [11]. Smokers tend to have an unhealthier lifestyle than never smokers and are more satisfied with a lower functional level, possibly distorting the test results [37]. Furthermore, smokers tend to receive primary TKA at a younger age than never smokers. As smokers generally tend to report higher pain scores than never smokers, the younger, actively smoking patients may achieve—owing to their better health status than older never smokers requiring TKA—higher functional ratings before TKA as well as during follow-up [22, 38].

The findings of this study indicate an overall beneficial effect of TKA on functional outcome regardless of smoking status, represented in a nearly even distribution of PROMs across all smoking status groups. However, as PROMs prior to surgery were not available, the authors could not evaluate whether smoking status groups respond differently to TKA.

The following limitations must be underlined: The retrospective design of the study produces a low level of evidence, as does the limited number of smoking patients, thus eventually reducing the generalization and reproducibility of results obtained. A risk analysis regarding the number of cigarettes smoked per day was not possible due to the algorithm during initial assessment only revealing smoking status. The percentage of smokers could have been underestimated, as patients could have defined themselves as a non-smoker either due to having stopped smoking or smoking cigarettes occasionally. It is possible that the percentage of patients who had to be revised and were smokers is overestimated as patients tend to start smoking in adolescence, but more likely stop smoking with growing age rather than starting it [39, 40]. The limited patient number impairs the comparability of the SSI rate obtained in comparison with the literature. Yet, an advantage of information obtained from the present cohort can be seen in its uniformity. As a benefit we want to mention that all complications had to be recorded due to the authors’ healthcare system, which allows reimbursement after adequate classification of the diagnosis-related groups only.

## Conclusion

Although the overall revision risk was not significantly higher in active smokers, soft-tissue complications were significantly more common in active smokers than never smokers. The slightly better results observed for smokers regarding functional outcome after primary elective TKA warrant further research to define the significance. We strongly recommend surgeons to advise their patients toward quitting smoking to maximize the success of primary TKA and minimize complication risks. The present findings underline the detrimental effects of smoking on postoperative complications, and ongoing research on the effects of nicotine abuse in orthopedic patients further strengthens the stance against smoking.

## Data Availability

Data available on request from the authors.
